# Mitochondrial DNA variants in the pathogenesis and metabolic alterations of diabetes mellitus

**DOI:** 10.1016/j.ymgmr.2024.101183

**Published:** 2024-12-28

**Authors:** Praveen Kumar K.S., M.N. Jyothi, Akila Prashant

**Affiliations:** a Department of Medical Genetics, JSS Medical College and Hospital, JSS-AHER, Mysuru 570015, India; b Department of Biochemistry, JSS Medical College and Hospital, JSS-AHER, Mysuru 570015, India; c SIG-TRRG, JSS Medical College and Hospitals, JSS-AHER, Mysuru - 570015, India

**Keywords:** Mitochondrial DNA, ATP production, Genetic variation, Biomarkers, Metabolic disorder

## Abstract

Mitochondrial DNA (mtDNA) variants considerably affect diabetes mellitus by disturbing mitochondrial function, energy metabolism, oxidative stress response, and even insulin secretion. The m.3243 A > G variants is associated with maternally inherited diabetes and deafness (MIDD), where early onset diabetes and hearing loss are prominent features. Other types of mtDNA variants involve genes ND4 and tRNA Ala genes that increase susceptibility to type 2 diabetes. Understanding these variants will provide a basis for developing targeted therapy to improve mitochondrial function and metabolic health. This article reviews the impact of mtDNA variants in diabetes, specifically with regards to the m.3243 A > G variant effects on mitochondrial function and insulin secretion and other mtDNA variants that contribute to diabetes susceptibility, particularly ND4 and tRNA Ala gene variants. Data from extant literature were synthesised to obtain an understanding of how mtDNA variants affect diabetes pathogenesis. The main defect for MIDD is the m.3243 A > G variant, which comprises enhanced susceptibility to metabolic syndrome and type 2 diabetes, followed by mitochondrial dysfunction, insulin resistance, and beta-cell dysfunction. Other mtDNA variants have also been reported to enhance diabetes susceptibility through mitochondrial dysfunction and insulin resistance. Increased production of reactive oxygen species (ROS) resulting from mitochondrial malfunction adds to metabolic and tissue damage. This happens in tissues crucial to glucose homeostasis, and it represents an important contribution of mitochondrial dysfunction to metabolic disturbances in diabetes. These mechanisms would underlie the rationale for developing targeted therapies to preserve mitochondrial function and, hence improve the metabolic health of diabetic patients.

## Highlights

• The key mutations in mtDNA are m.3243A > G. Such mutations compromise mitochondrial function due to disturbances in insulin secretion, making people more likely to develop type 2 diabetes and MIDD.
• Mechanisms include disrupting average cellular energy production and managing oxidative stress, such as intracellular calcium signaling with subsequent metabolic dysfunction and insulin resistance.
• mtDNA mutations are also predisposed to complications, including diabetic nephropathy, retinopathy, and cardiovascular complications, which wear off the severity of diabetes. Research Models Animal and cell culture models have identified molecular pathways affected by mtDNA mutations, thus providing potential targets for novel diabetes treatments.
• Understanding the role of mtDNA in diabetes not only sheds light on the disease's mechanisms but also opens up avenues for targeted therapy. This could significantly improve the function and metabolic health of mitochondria, offering hope for more effective diabetes treatments.

## 1 Introduction

Diabetes mellitus, a metabolic disorder characterized by elevated blood glucose levels, arises from defects in insulin secretion, insulin action, or both [1]. The condition is classified into several types, with type 1 diabetes resulting from autoimmune destruction of pancreatic beta cells, leading to insulin deficiency, and type 2 diabetes stemming from a combination of insulin resistance and inadequate insulin secretion [2]. Other forms include gestational diabetes, which occurs during pregnancy, and monogenic diabetes, caused by variants in a single gene [3]. Mitochondria, the cellular organelle responsible for energy production, play a crucial role in glucose metabolism. These organelles generate Adenosine triphosphate (ATP) through Oxidative phosphorylation (OXPHOS), a process involving the electron transport chain. In addition to energy production, mitochondria regulate various cellular processes, including calcium signaling, apoptosis, and ROS production [4]. Dysfunction in these organelles can disrupt cellular homeostasis and contribute to the development of metabolic disorders like diabetes [5]. Mitochondrial DNA, a small circular genome separate from the nuclear DNA, encodes essential genes for mitochondrial function, including those involved in OXPHOS. Variants in mtDNA can impair mitochondrial function, leading to decreased ATP production, increased ROS production, and altered cellular metabolism [6,7]. In the context of diabetes, mtDNA variants have been implicated in both the pathogenesis of the disease and the development of its complications [8]. Studies have shown that specific mtDNA variants, such as the m.3243 A > G variant, are associated with an increased risk of gestational diabetes and pregnancy complications in women [9]. Additionally, mitochondrial dysfunction in peripheral blood cells has been linked to poor glycemic control and lower bone mineral density in individuals with diabetes [10]. Stem cell culture studies have further highlighted the impact of mtDNA varinats on cellular and molecular alterations, providing insights into potential therapeutic strategies. These variants can serve as models for studying variant effects and sources for cell-replacement therapy, offering promising avenues for future research in diabetes treatment [11].

Understanding the role of mtDNA variants in diabetes is crucial for developing targeted therapies and interventions [12]. This review aims to provide a comprehensive overview of the current knowledge regarding mtDNA variants in diabetes, focusing on their types, prevalence, impact on mitochondrial function and cellular metabolism, and their contribution to the development of diabetes-related complications. Furthermore, the review will explore the diagnostic and prognostic implications of mtDNA variants in diabetes. It will discuss the potential use of mtDNA analysis as a biomarker for disease risk stratification and monitoring. Emerging therapeutic strategies targeting mitochondrial dysfunction in diabetes will also be discussed, along with the challenges and opportunities in translating these findings into clinical practice. The objective of this review is to contribute to the mitochondrial biology and diabetes pathophysiology. By elucidating the role of mtDNA variants in diabetes, the review aims to pave the way for the development of novel therapeutic approaches to mitigate the impact of mitochondrial dysfunction in diabetes. Understanding the molecular mechanisms linking mtDNA variants to diabetes is crucial for developing targeted therapies and improving patient outcomes.

### 1.1 Mitochondrial metabolic function and dysfunction

Mitochondria play an important role in cellular metabolism, particularly in energy production and various metabolic pathways. Here are some key aspects of mitochondria's metabolic functions like Energy Production, Beta-Oxidation, Citric Acid Cycle (Krebs Cycle), Metabolite Shuttle, Calcium Homeostasis, Heme Biosynthesis, ROS Production and Antioxidant Defense, Apoptosis etc., [13]. The dysfunction of mitochondria can lead to various metabolic disorders, including diabetes, neurodegenerative diseases, and cardiovascular diseases [14]. Studies by Markin et al. evaluated the role of mitochondrial dysfunction in chronic human disorders like atherosclerosis and diabetes mellitus. It highlights the importance of mitochondrial turnover processes, such as fission, fusion, and mitophagy, in maintaining a functional mitochondria population in cells. Dysfunction in these processes can lead to the accumulation of dysfunctional mitochondria, deficient energy production, increased oxidative stress, and cell death. Mitochondrial dysfunction in atherosclerosis, with mtDNA variants contributing to the formation of atherosclerotic lesions. Declining mitochondrial function contributes to aging processes and age-related diseases. Further, white adipose tissue to increase energy expenditure, which is being studied for its effects on metabolic diseases like diabetes and atherosclerosis [15]. Studies by Baishali Alok Jana et al. examines the link between cytosolic lipid excess, mitochondrial dysfunction, and insulin resistance in skeletal muscle, especially in the context of high-fat diet-induced metabolic disturbances. Shows that excess lipids accumulate in tissues and can negatively impact glucose homeostasis. Although mitochondrial dysfunction is observed in type 2 diabetes mellitus, it is unclear whether it is the cause or result of insulin resistance and explores the molecular pathways linking cytosolic lipid excess and mitochondrial dysfunction, including reactive oxygen species, aging, and decreased mitochondrial biogenesis in skeletal muscle [16]. Mitochondrial diabetes (MD), a rare form of monogenic diabetes, is primarily characterized by insulin secretion failure in pancreatic β-cells due to impaired mitochondrial ATP production. However, it has also been linked to insulin resistance (IR), a clinical feature. Mitochondrial dysfunction is a key factor contributing to IR, suggesting a dual role of mitochondria in MD. The review discusses the insulin signaling and molecular mechanisms of IR, emphasizing the importance of mitochondria in cellular glucose metabolism and insulin secretion. It also discusses confirmed pathogenic mtDNA variants were associated with diabetes, particularly those in the transfer RNA (tRNA) genes of the mitochondrial genome. The article suggests that mitochondrial dysfunction in MD may not only lead to impaired ATP production but also contribute to IR, possibly through aberrations in taurine modifications in tRNA genes. The article emphasizes the critical role of mitochondria in diabetes development and the need for further research to understand the precise mechanisms underlying mitochondrial dysfunction in relation to IR and diabetes [17].

Studies by Cagla Cömert et al. studied the effects of two mtDNA variants, T10609C and C10676G, on the proton translocation mechanism in the mitochondrial respiratory complex I. The variants disrupted the proton translocation pathway, leading to mitochondrial dysfunction and increased reactive oxygen species production. The study suggests these varinats could serve as specific genetic biomarkers for type 2 diabetes mellitus (T2DM) and cataracts, with a computational assay potentially validating these biomarkers [18]. The homologous recombination deficiency (HRD) and its impact on PARP inhibitors (PARPi) showed that HRD cancer cells shift from glycolytic to oxidative metabolism, relying on oxidative phosphorylation (OXPHOS) for DNA repair. This adaptation makes them more sensitive to metformin and NAD+ levels. Conversely, high glycolytic metabolism reduces PARPi effectiveness. The study suggests that cancer cell metabolic state, particularly OXPHOS reliance, can affect their response to PARPi, suggesting potential strategies for improving PARPi effectiveness in HRD cancers [19]. Studies also showed that variants in mitochondrial and nuclear genes can lead to dysfunction. As mitochondria play a crucial role in regulating β-cell insulin secretion, and dysfunction in this process is a hallmark of diabetes. Abnormalities in mitochondrial fission and fusion dynamics are linked to the disease, as shown by altered levels of certain proteins in diabetic patients. Further demonstrated that the Nr4a family of orphan nuclear receptors is essential for mitochondrial function, and its overexpression can induce pancreatic β-cell proliferation. Impaired oxidative phosphorylation (OXPHOS) function contributes to β-cell dysfunction in insulin resistance infers primary mitochondrial diseases are rare, mitochondrial defects are associated with common diseases like diabetes (H. [20]). Non-synonymous mtDNA variants in the *MT-ND1* gene, which is found in patients with diabetes. The variants caused dysfunction in complex I of the mitochondrial respiratory chain, resulting in decreased activity and quantity. This suggests that mitochondrial dysfunction could contribute to the development of diabetes, which often results from an imbalance in glucose metabolism and insulin secretion. The findings offer insights into the potential link between mitochondrial dysfunction and diabetes, emphasizing the importance of understanding molecular mechanisms underlying mitochondrial defects in diabetes pathogenesis. These insights highlight the need for targeted therapies aimed at improving mitochondrial function, which could play a crucial role in preventing or treating diabetes [21].

### 1.2 mtDNA variants linking diabetes in different cohorts

In different cohorts, variants in mtDNA have been linked to the pathophysiology of diabetic mellitus (DM). The mtDNA A10398G polymorphism in the Asian Indian Cohort is hypothesized to affect mitochondrial function, which in turn causes beta-cell malfunction and insulin resistance. It is found in the *ND3* gene of complex I of the electron transport chain (ETC). The mtDNA 16,189 T > C variation has been linked to a higher incidence of type 2 diabetes. This variant, which is found in the mtDNA D-loop region, may have an impact on transcription and replication of mtDNA, which could impair glucose metabolism and cause mitochondrial malfunction in Chinese people. mtDNA haplogroup J has been linked to a higher risk of type 2 diabetes, whereas haplogroup H has been linked to a lower risk. It is believed that these haplogroups affect European mitochondrial function and energy metabolism. An elevated risk of type 2 diabetes has been linked to the tDNA 5178 A > C polymorphism in the *ND2* gene. It has been demonstrated that this polymorphism reduces oxidative stress and impairs mitochondrial function, which may help Japanese and A higher risk of diabetes and its complications has been linked to the *MT-TL1* gene mtDNA 3243 A > G variant. In Africans, this variant impairs the function of mitochondrial tRNA, which results in decreased mitochondrial protein synthesis and energy generation. Further, many studies showed mtDNA linked to diabetes in different populations.

The Study by Freeney et al. examined the impact of the m.3243 A > G variant on pregnancy outcomes in women with mitochondrial disease. The study involved 67 women with confirmed mitochondrial disease and 69 unaffected women. Results showed that pregnancies with the m.3243 A > G variant had higher rates of gestational diabetes, breathing difficulties, and hypertension. Only half of these pregnancies had normal vaginal delivery, with many requiring emergency caesarean section. Babies born to mothers with the m.3243 A > G variant were born earlier and had lower birthweights. Over half of these babies were preterm and required resuscitation and admission to the special care baby unit. The study concluded that women with the m.3243 A > G variant are at a higher risk of complications during pregnancies, including caesarean section and preterm delivery, highlighting the need for specialized care for pregnant women with mitochondrial disease [22] The m.3243 A > G variant in mitochondrial DNA, function, and clinical severity in individuals with diabetes found significant mitochondrial dysfunction in peripheral blood mononuclear cells and was associated with worse glycemic control and lower bone mineral density. The study emphasizes the importance of mitochondrial function in diabetes and suggests measuring heteroplasmy levels and assessing mitochondrial membrane potential could help evaluate the severity [23].

The study by Tabebi et al. focuses on mitochondrial diabetes (MD), a rare form of monogenic diabetes characterized by familial clustering and bilateral hearing impairment in carriers. The most common form of MD is associated with the m.3243 A > G variant in the mitochondrial *MT-TL1* gene, but other variations, deletions, and depletion in mtDNA are also linked to MD. The study found a family with clinical features of MD, including cardiomyopathy, retinopathy, and psychomotor retardation. The proband had the m.3243 A > G variant in a heteroplasmic state, a large mtDNA deletion, and a reduction in mtDNA copy number. This variant affects oxidative phosphorylation and ATP production, leading to mitochondrial dysfunction. Other mitochondrial variations in the proband, such as substitutions in the MT-ND1 and *MT-ND6* genes, are associated with diabetes and deafness. The study highlights the complex phenotype associated with the m.3243 A > G variant and the importance of mitochondrial genome anomalies in MD [24] studies by Yagil et al. examined the role of mitochondria in diabetes development using the Cohen diabetic rat model. They identified a variant in the *NDUFA4* gene, which leads to the absence of the NDUFA4 protein, affecting mitochondrial function. The study found reduced activity in mitochondrial complex I and complex IV activity in diabetic rats exposed to a diabetogenic diet, indicating an impact of the diet on complex IV unrelated to the gene variation. ATP levels failed to increase in diabetic rats in response to the diet, suggesting that the variant combined with the metabolic strain prevented the expected increase in ATP production, which is essential for insulin secretion from the pancreas. The diabetic rats also showed elevated oxidative stress, indicating that the mitochondrial dysfunction caused by the variation, coupled with the metabolic strain of the diet, led to increased oxidative stress, impairing insulin secretion and contributing to diabetes development [25].

### 1.3 Gestational diabetes mellitus (GDM) and mitochondrial dysfunction

The relationship between mitochondrial dysfunction and GDM is increasingly observed since maternal metabolic burden affects mothers' and babies' health. Mutations in mtDNA have been proven to cause impaired mitochondria function in pregnant women, which might predispose the latter to GDM due to disruption in glucose metabolism and production of cellular energy; interference in insulin signaling is an oxidative stress event that leads to the condition of insulin resistance characteristic of GDM.

A study by Alharbi et al. investigated the link between GDM in Saudi women and common mitochondrial variations. The researchers conducted a case-control study with 96 GDM and 102 non-GDM pregnant women, analyzing DNA for these genetic variations. They found no heteroplasmy or homozygous variations in any subjects, indicating that these variations do not play a role in GDM in Saudi women. GDM is characterized by glucose intolerance during pregnancy, linked to oxidative stress and mitochondrial dysfunction. The study suggests further meta-analyses are needed to better understand the global relationship between GDM and mitochondrial variations [26]. Zhang et al.'s study explores the role of TRAP1 in inhibiting MARCH5-mediated degradation of MIC60, a protein essential for mitochondrial cristae structure, to protect against mitochondrial dysfunction and apoptosis in cardiomyocytes under diabetic conditions. The researchers found that high glucose/palmitate conditions led to mitochondrial dysfunction and cell apoptosis, with decreased MIC60 levels due to ubiquitination and degradation. Exogenous expression of MIC60 improved cristae structure, mitochondrial function and reduced apoptosis. Disrupting the interaction between MIC60 and MARCH5 or silencing MARCH5 prevented MIC60 degradation and alleviated dysfunction. TRAP1, a mitochondrial chaperone, inhibited MIC60 ubiquitination by competing with MARCH5 for binding [27]. The study by Kim et al. explores the role of zinc transporter 8 (ZnT8) in type 1 diabetes (T1D). The researchers found that complete loss of ZnT8 accelerated T1D onset, while heterozygosity partially protected it. ZnT8 deficiency led to more rampant autoimmunity, suggesting a role in regulating immune responses. The study also found that altering intra-islet zinc homeostasis to regulate mitochondrial respiration may be a novel way to modulate T1D pathophysiology [28]. Studies by Schartnera et al., found that high glucose levels reduced SIRT2 expression, leading to impaired mitochondrial function and suppressed neurite outgrowth in sensory neurons. Inhibiting the polyol pathway with specific inhibitors restored SIRT2 expression and improved mitochondrial function, suggesting that enhancing SIRT2 signaling could be a potential therapeutic strategy for diabetic neuropathy [29].

### 1.4 Mitochondrial DNA variations in the pathogenesis of diabetes

Impaired mitochondrial function is an excellent issue in energy metabolism and cellular homeostasis due to its critical role in the pathogenesis of diabetes through mtDNA variants. The m.3243 A > G variant, which usually results in maternally inherited diabetes and deafness, increases insulin resistance and dysfunction in beta cells [30]. The m.3243 A > G variant affects only mitochondrial protein synthesis, resulting in defective adenosine triphosphate (ATP) production and increased reactive oxygen species (ROS) (D. [31]). These aberrant mitochondrial bioenergetics may cause metabolic derangements that predispose to type 2 diabetes, establishing the key role of mtDNA variants in diabetic pathophysiology (M. [32]).

Adding more complexity to this scenario, other variants in mtDNA in genes such as ND4 and tRNA Ala contribute to the overall diabetes susceptibility through different mechanisms. Variants in ND4 have, for example, been linked to the generation of excess ROS that diminishes the mitochondrial membrane potential, resulting in impaired insulin secretion [33]. Similarly, variants in tRNA can disrupt mitochondrial protein synthesis, which is critical for maintaining the integrity of the electron transport chain and efficient ATP generation [34]. This reveals that mtDNA variants contribute to diabetes pathogenesis through multifaceted mechanisms and thus the need to include not only direct effects on insulin secretion but also broader influences on cellular metabolism in the role of mitochondrial dysfunction in the development of diabetes. [35].

#### 1.4.1 Mitochondrial dysfunction may contribute to insulin secretion and insulin resistance

Both insulin secretion and resistance are essential events in the onset of diabetes, and mitochondrial dysfunction is also involved [17]. In the pancreatic beta-cells, the process of insulin secretion has to be regulated based on ATP production, which drives the exocytosis of insulin granules [10]. The m.3243 A > G variant had been shown to decrease ATP production by mitochondria to levels that were insufficient enough to directly lower insulin secretions [36].

Further, the m.3243 A > G variant disrupts calcium signaling essential for the exocytosis of insulin from beta-cells, which aggravates the disturbance of glucose homeostasis. [37]. This not only impairs the short-term response of the beta-cell to glucose but also contributes to long-term dysfunction of the beta-cell that worsens the hyperglycemic condition characteristic of diabetes [38]. On the other hand, most variants of mtDNA, particularly in the genes that code for ND4 and tRNA, cause significant contributions to the insurgence of insulin resistance. It is related to the malfunction of mitochondria involving an enhanced level of production of ROS and also to peripheral tissue-initiated insulin resistance [39]. In particular, increased ROS can damage insulin signaling pathways, thus impairing the action of insulin to induce glucose uptake by muscle and adipose tissues [40]. As a consequence, there are evident differences between various mtDNA variants concerning their effects on the pathophysiology of diabetes. For instance, some of them are involved mainly in the impairment of insulin secretion, whereas others participate in the manifestation of insulin resistance [41,42]. Understanding these other mechanisms would lead to designing targeted therapeutic approaches aimed at recovering mitochondrial function and markedly reducing the impact of these variants in diabetes pathogenesis [43]. To conclude, the variants of mtDNA are of great importance in the pathogenesis of diabetes. They work to impair insulin secretion and mechanisms related to insulin resistance. In particular, the role of the variant m.3243 A > G, ND4, and tRNA Ala in the pathogenesis of diabetes explains complications of mitochondrial dysfunction in connection with metabolic regulation. Hence, further research on such mechanisms is essential in discovering new therapeutic targets that can help improve the outcomes in treating patients with diabetes. Elucidation of the distinct contributions of such variants can help understand a usually complex interaction between mitochondrial health and diabetes.

### 1.5 mtDNA variants linking complications of diabetes

Mitochondrial abnormalities play a significant role in the development of complications in diabetes, especially microvascular and macrovascular issues. Mutations in mtDNA, such as those found in genes MT-TL1 and ND5, have been shown to exacerbate conditions like diabetic nephropathy, retinopathy, and peripheral neuropathy by impairing oxidative phosphorylation (OXPHOS) and increasing ROS production. These effects compromise vascular integrity and cellular energy supply, exacerbating the progression of diabetes complications.

Several studies have investigated the role of mtDNA variants in the development and progression of complications associated with diabetes mellitus (DM) specially causing microvascular and macrovascular complications viz., mtDNA 3243 A > G Variant (*MT-TL1* Gene), mtDNA 8993 T > G Variant (*ATP6* Gene), mtDNA 13513G > A Variant (*ND5* Gene), mtDNA Deletions and mtDNA haplotypes. Here we summarized the studies related to mtDNA linking different complications in diabetes.

The study by Gazdikova et al. examines the role of mitochondria in kidney diseases, specifically chronic kidney disease (CKD) and acute kidney injury (AKI) in diabetes. Mitochondria are crucial for energy production, redox signaling, and inflammation, and their dysfunction is linked to various kidney diseases. The study reveals that mitochondrial defects in CKD contribute to tubular syndromes, interstitial nephritis, focal and segmental glomerulosclerosis, and diabetic nephropathy. Mitochondrial dysfunction in CKD contributes to muscle weakness and atrophy, a condition known as “acquired mitochondrial myopathy” and uremic sarcopenia. In AKI, mitochondria serve as a source of energy and a regulator of cell death, leading to tubular injury and persistent renal insufficiency. The study suggests that understanding mitochondria's role in kidney diseases could improve diagnostics and therapies for patients with mitochondrial nephropathies or renal damage [44].

A 57-year-old Japanese man with MIDD developed chronic kidney disease (CKD) due to delayed diagnosis. Renal biopsies revealed mitochondrial degeneration in tubular epithelial cells and accumulating mitochondria in podocytes and tubular cells, indicating mitochondrial dysfunction. Despite good glycemic control and no hypertension, the patient developed nephrosclerosis over 20 years. The absence of typical diabetic nephropathy lesions suggested mitochondrial cytopathy contributed to CKD progression. Treatment with taurine supplementation was initiated to prevent further deterioration. The case highlights the importance of considering mitochondrial dysfunction in patients with atypical diabetes-related complications, especially when kidney disease is involved [45]. A study by Ryang Na et al. examined the role of mitochondrial oxidative phosphorylation (OxPhos) in podocyte dysfunction, a common symptom of primary and diabetic glomerular diseases. They used a mouse model with a *CRIF1* gene loss of function variant, which is crucial for OxPhos polypeptide production and insertion into the mitochondrial membrane. The results showed that CRIF1 deficiency in podocytes led to mitochondrial dysfunction, structural abnormalities, and profound albuminuria, which progressed to glomerular sclerosis and interstitial fibrosis by 20 weeks. The study underscores the importance of mitochondrial OxPhos function in maintaining podocyte integrity [46]. The study by Wang et al. found that mitochondrial dysfunction is a significant factor in podocyte injury, which is a key factor in proteinuria in diabetic nephropathy. The study found that Cdk5 expression and activity are upregulated in diabetic conditions, leading to podocyte injury characterized by decreased synaptopodin and nephrin expression and structural and functional mitochondrial dysfunction. Inhibition of Cdk5 attenuated podocyte injury and improved mitochondrial function by reducing ROS levels, decreasing cytochrome *c* release, and increasing ATP production. The study also highlighted the role of Cdk5-mediated Sirt1 phosphorylation in mitochondrial dysfunction and podocyte injury in diabetic nephropathy, suggesting that targeting the Cdk5-Sirt1 signaling pathway could be a potential therapeutic strategy [47].

The study explores the link between mtDNA heteroplasmy, mitochondrial function, and clinical severity in individuals with peripheral artery disease (PAD), particularly diabetes as a comorbidity. The researchers hypothesized that individuals with diabetes and PAD would have higher levels of mtDNA heteroplasmy, particularly in regions susceptible to oxidative damage, which would correlate with ischemia and mobility impairment. The study found that individuals with PAD and diabetes had higher levels of “low frequency” heteroplasmy in their muscle, particularly in regions like the displacement loop. Lower mitochondrial damage, as indicated by low mtDNA copy number and microheteroplasmy, was associated with better walking performance in PAD patients with diabetes [48]. A study by Oishi et al. examined a patient with MIDD due to a mitochondrial DNA variant. The patient had sensorineural hearing loss, diabetes mellitus, and macular dystrophy. High-resolution imaging techniques revealed that primary photoreceptor dysfunction in the fovea might precede retinal pigment epithelium dysfunction in MIDD, suggesting mitochondrial dysfunction in photoreceptors could contribute to macular dystrophy. The study underscored the importance of whole exome sequencing in detecting mitochondrial DNA variants in MIDD [49]. The study by Peppel et al. discusses the heterogeneity in clinical phenotypes in MIDD and mitochondrial encephalomyopathy with lactic acidosis and stroke-like episodes (MELAS) diseases, which are attributed to varying levels of heteroplasmy in different tissues. High heteroplasmy levels in the brain lead to MELAS, while high levels in the pancreas result in diabetes mellitus. Studies show that brain tissue of symptomatic MELAS patients consistently displays heteroplasmy levels of >70 %. However, correlating these levels with clinical outcomes requires caution due to variability and decline with age. The severity and progression of MIDD are likely affected by factors like sex, environmental factors, and genetic modifiers. Future research is needed to develop a more comprehensive model to predict MIDD phenotype severity and progression [50]. Studies by Katie Nahay Robinson et al. discuss MIDD, a rare diabetic syndrome caused by a point variant in mitochondrial DNA. MIDD is primarily inherited through the maternal oocyte and can cause diabetes mellitus, deafness, and organ-specific complications. Managing MIDD requires a multidisciplinary approach, including screening for comorbidities, selecting appropriate therapies, and offering genetic counselling. Treatment strategies address mitochondrial dysfunction and insulin deficiency, with insulin secretagogues being the preferred oral glucose-lowering agents. Retinal manifestations in MIDD differ from traditional diabetes-related complications, with patients often developing macular dystrophy and retinal lesions [51].

A study by Sahara et al. reported a patient with severe bilateral sensorineural hearing loss (SNHL) who underwent cochlear implantation (CI) in her left ear. The A8296G variant, rare but associated with hearing loss, was found to be beneficial for the patient's hearing and speech discrimination. The findings underscore the significance of mitochondrial genetics in understanding hearing loss and its progression, suggesting that SNHL patients with this variant may benefit from CI [52]. Studies by Saku et al., examined three cases of mitochondrial cardiomyopathy with the m.3243 A > G variant, revealing cardiac hypertrophy, juvenile-onset diabetes mellitus, and hearing loss. The severity of cardiac involvement and duration of symptoms varied among cases. Histological analysis showed mitochondrial degeneration might correlate with impaired heart function in patients with mitochondrial cardiomyopathy, suggesting mitochondrial degeneration contributes to heart failure progression [53]. Guodong Pan et al. conducted a study on Empagliflozin (EMP), a sodium-glucose cotransporter 2 inhibitor, and its impact on diabetic cardiomyopathy in ALDH2 * 2 mutant mice. The mice, which are a model for East Asians with the E487K variant in ALDH2, were induced with type-2 diabetes and treated with EMP for two months. The study found that EMP improved cardiac function and exercise performance, attributed to its ability to decrease protein adducts in cardiac mitochondria and increase AKT-AS160-GLUT-4 signaling in skeletal muscle [54]. The study investigates the role of Protein kinase R-like endoplasmic reticulum kinase (PERK) in cardiac valve formation in Wolcott-Rallison syndrome, a condition characterized by early-onset diabetes mellitus. The researchers found that PERK inhibition suppressed mitochondrial metabolic activity by affecting fatty acid oxidation and endocardial-mesenchymal transformation. This suggests that PERK signaling is crucial for cardiac valve formation, highlighting the role of mitochondrial function in diabetes-related cardiac complications [55].

## 2 Diabetic retinopathy

Diabetic retinopathy (DR) is a leading microvascular complication of diabetes mellitus, characterized by damage to retinal blood vessels, which can lead to vision loss. Retinal cells, due to their low oxygen demands compared to other tissues, may exhibit slower progression in some cases of DR. This hypothesis, as discussed in the literature [56], highlights the complex interplay of mitochondrial dysfunction and oxidative stress in retinal pathology. Few studies highlight that the unique retinal microvascular environment, with its low oxygen extraction, contributes to delayed signs of vascular dysfunction compared to other tissues. Optical coherence tomography angiography (OCTA) has demonstrated subtle microvascular abnormalities, such as reduced perfusion density and vessel density, which precede overt diabetic retinopathy. Further, systemic factors such as chronic kidney disease, dyslipidemia, and hyperglycemia significantly influence retinal microvascular changes. This is particularly important for understanding why diabetic retinopathy might progress differently based on individual systemic conditions. So, the lower oxygen demands aligns with findings of reduced metabolic stress in certain retinal regions, possibly delaying the onset of clinical symptoms [57].

Studies by Manish Mishra et al. reveal that mitochondrial DNA methylation and base mismatches are crucial in the development of diabetic retinopathy. In diabetic retinopathy, retinal mitochondria display dysfunction, and mtDNA is damaged due to increased base mismatches and hypermethylated cytosines, particularly in the displacement loop region. The researchers inhibited DNA methylation and regulated cytosine deamination factors using human retinal endothelial cells and a diabetic mouse model. They found that inhibiting DNA methylation or regulating deamination factors significantly reduced base mismatches at the D-loop and prevented mitochondrial dysfunction. This crosstalk persists even after hyperglycemia termination, suggesting a role in the metabolic memory phenomenon associated with diabetic retinopathy progression [58].

Mitochondrial dysfunction contributes significantly to the pathogenesis of DR by promoting reactive oxygen species (ROS) production, leading to oxidative damage and inflammation. Key mtDNA variants, such as m.3243 A > G, are linked to impaired mitochondrial function in retinal cells, exacerbating vascular leakage and neovascularisation [59]. Emerging research underscores the role of mitochondrial epigenetic changes, including mtDNA methylation, in perpetuating the “metabolic memory” phenomenon, which sustains retinal damage even after achieving glycemic control [60]. Understanding these mechanisms can aid in identifying therapeutic targets to prevent or mitigate DR.

## 3 Macrovascular complications

Mitochondrial dysfunction in diabetes also extends to macrovascular complications, which include cardiovascular diseases (CVD), atherosclerosis, and peripheral arterial disease [61]. These complications are significant contributors to morbidity and mortality in diabetes patients [62]. Impaired mitochondrial oxidative phosphorylation (OXPHOS) disrupts ATP production, leading to endothelial dysfunction, increased ROS generation, and chronic inflammation—key drivers of atherosclerosis [63].

Studies have identified specific mtDNA variants, such as those in the ND4 and ND5 genes, which enhance susceptibility to atherosclerotic plaque formation by promoting oxidative damage [64]. Mitochondrial dysfunction also alters lipid metabolism, resulting in lipid accumulation and foam cell formation within vascular walls. [65]. These processes underscore the systemic impact of mitochondrial abnormalities in diabetes and highlight the need for integrated therapeutic strategies targeting mitochondrial health to combat macrovascular complications.

### 3.1 Animal studies related to mtDNA variants

**Fig. 1:**
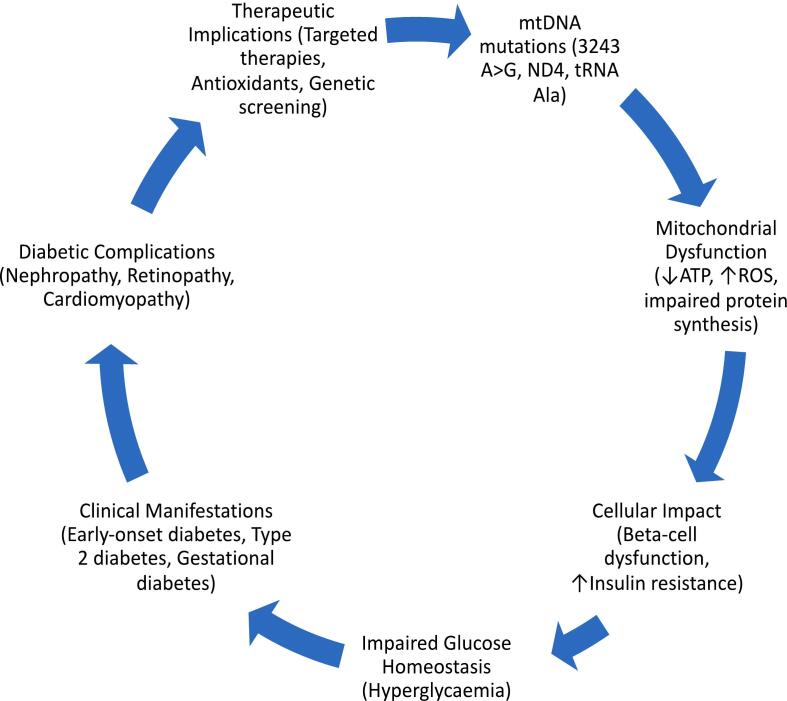
Schematic representation of mtDNA variants linking Diabetes Mellitus.

Animal studies provide valuable insights into the impact of these variants on mitochondrial function and disease. mtDNA variants linking diabetes studied mostly in *CRIF1* Deficiency in Mice, Cdk5 Expression in Diabetic Nephropathy, ALDH2*2 Mutant Mice, Mitochondrial Cardiomyopathy in Mice. Showed that the animal studies would be of greater importance to understand the pathophysiology and mechanistic role of mtDNA variants in diabetes. Here we listed studies which had insights in understanding the molecular pathway by through animal models. Studies were tabulated in Table 2. (See Fig. 1.) (See Table 1.)

**Table 2:** Animal studies showing the impact of mtDNA variant linking diabetes.

Study and Reference	Variant	Animal Model	Impact
Adams et al. [66]	CRIF1 Deficiency	NOD mouse model	Mitochondrial dysfunction may influence T1D pathogenesis, leading to novel therapeutic avenues
Dumesic et al. [67]	uORF variant in *PPARGC1A* gene	Mouse model	Increased PGC1a protein levels, enhanced oxidative metabolism, and protection from acute kidney injury, implications for diseases like diabetes, neurodegeneration, cancer, and kidney disease
Herbst et al. [68]	N/A	Older rats	Metformin increased mtDNA copy number, reduced cardiac mass, and lowered mitochondrial complex I-dependent respiration in the heart
Kunath et al. [69]	*Nnt* gene truncation	C57BL/6 J mice	Wild-type mice were more sensitive to diet-induced obesity and had higher relative fat mass
Y. Li et al. [70]	N/A	GK rats	Hyperglycemia activates Cdk5, leading to AMPK-a2Thr485 phosphorylation and inhibition of AMPK-a2 activity in the hippocampus, reduced neuron proliferation and viability
Liu et al. [71]	N/A	Mouse model	Ethoxyquin (EQ) treatment improved nerve conduction and reduced mtDNA deletions in the sciatic nerve of diabetic mice
McCrimmon et al. [72]	Partial deletion of CrAT	Heteroplasmy mouse model	Importance of intact mitochondrial substrate efflux in preventing kidney disease, sex differences in response to mitochondrial dysfunction
Pan et al. [73]	ALDH2*2 variant	ALDH2*2 mutant mice	ALDH2 activation protects against 4HNE-induced coronary endothelial cell injury and cardiac dysfunction
Pittala et al. [74]	N/A	Mouse model	VDAC1-based peptide restored blood glucose levels, increased islet size and number, improved insulin content
Tang et al. [75]	MPV17 deficiency	Mpv17-deficient mice	MPV17 acts autonomously to promote β-cell apoptosis, resistance to diabetes induced by β-cell loss and apoptosis
Wang et al. [76]	N/A	Type II diabetes mice	Irisin treatment improved insulin sensitivity, glucose tolerance, preserved cardiac function, linked to activation of the p38 pathway and reduction of HDAC4
Tate et al. [77]	N/A	Akita mice	MitoGamide improved markers of left ventricular diastolic dysfunction in diabetic mice, targeting mitochondrial dysfunction
Yagil et al. [25]	*NDUFA4* gene deletion	Diabetic rats	*NDUFA4* gene deletion caused reduced mitochondrial complex I and IV activities, decreased ATP production, and increased oxidative stress
Turchi et al. [78]	*FXN* gene variant	Murine model (KIKO)	FXN deficiency in vWAT led to adipocyte expansion, hypovascularization, pro-inflammatory adipokines, immune cell recruitment, and fibrosis, similar to type 2 diabetes

**Table 3:** Cell culture studies linking mtDNA variants linking diabetes.

Study	Variant	Cell Model	Functional impact
Gao et al. [79]	m.3243A > G	Urine-derived stem cells (USCs)	Association with bone mineralization deficiency, potential for autologous cell-replacement therapy, identified ATF5 as potential therapeutic target
Mullin et al. [80]	m.3243A > G	Skin-derived dermal fibroblasts	Positive correlation between % m.3243G in fibroblasts and blood, negative correlation with mtDNA copy number, negative correlation between % m.3243G and age of onset of visual symptoms
Chae et al. [81]	m.3243A > G	Patient case	Challenges in surgical management due to higher risk of complications like multiorgan hypofunction, abnormal drug reactions, and perioperative death
Colclough et al. [82]	N/A	Bone marrow-derived mesenchymal stem/stromal cells (BM-MSCs)	Safe and well-tolerated in T2DM patients, short-term therapeutic effects observed in patients with T2DM duration <10 years and BMI <23, T2DM duration altered proliferation rate of BM-MSCs, compromised glycolysis and mitochondrial respiration, induced mtDNA variants accumulation
Jiang et al. [83]	Multiple mtDNA variants	Induced pluripotent stem cells (iPSCs)	Higher levels of homoplasmic variants in T2DM iPSCs, lower oxygen consumption rates, decreased insulin production, and reduced insulin secretion in response to glucose, mtDNA variants contribute to mitochondrial dysfunction in pancreatic cells
Shand et al. [84]	Multiple mtDNA variants	Induced pluripotent stem cells (iPSCs)	Higher levels of homoplasmic variants in T2DM iPSCs, lower oxygen consumption rates, decreased insulin production, and reduced insulin secretion in response to glucose, emphasize the importance of screening mtDNA variants before use in disease modeling or autologous cell therapy for diabetes
Zhang et al. [85]	N/A	Vascular smooth muscle cells (VSMCs)	Hydrogen sulphide (H2S) can inhibit vascular smooth muscle cell (VSMC) proliferation in hyperglycemia and hyperlipidemia, mechanism involves mitochondrial pyruvate dehydrogenase complex-E1 (PDC-E1), exogenous H2S inhibits PDC-E1 translocation by S-sulfhydration, reducing VSMC proliferation
Cömert et al. [14]	Negative HSP60 mutant	HEK293 cells	Oxidative stress and mitochondrial dysfunction contributing to neurodegenerative diseases, cancer, diabetes, and metabolic syndrome
Jennings et al. [86]	Recessive variants in DNAJC3	Patient fibroblasts	Dysregulation in lipid metabolism, mitochondrial bioenergetics, ER-Golgi function, and amyloid-beta processing, suggesting a link between mitochondrial dysfunction and diabetes

**Table 4:** Case/ familial studies on mtDNA variants linking diabetes.

Study Ref	Variant	Clinical Findings	Impact on Mitochondrial Function	Implications for Diagnosis and Management
Bai et al. [87]	m.3243A > G	Maternally inherited diabetes mellitus and deafness (MIDD); Diabetes and hearing loss in family members	Impaired mitochondrial protein synthesis and OXPHOS	Emphasizes the importance of genetic testing and physical examination in atypical diabetes presentations
Ding et al. [88]	ND4 G11696A, m.C5601T in tRNAAla, m.T5813C in tRNALys	Type 2 Diabetes (T2DM) in two Han Chinese families	Increased ROS production, decreased mitochondrial membrane potential, ATP, Complex I activity, NAD+/NADH ratio	Identifies specific mtDNA variants contributing to T2DM pathogenesis
Jiang et al. [89]	ND5 T12338C, tRNAAla T5587C	Maternally inherited T2DM in four out of seven relatives	Impaired mitochondrial function and increased diabetes susceptibility	Highlights mtDNA variants as potential contributors to T2DM in familial contexts
Fukuda and Nagao [90]	3243A > G	Overlapping MIDD and MELAS; Elevated GDF-15 levels during stroke-like episodes	Acute mitochondrial insufficiency exacerbation	Importance of monitoring GDF-15 and amino acid levels in patients with mitochondrial disease
Gustafson et al. [91]	De novo heteroplasmy variant in *SSBP1* gene	Severe mitochondrial disease symptoms, including diabetes	Large-scale mtDNA deletions due to altered DNA binding and decreased thermostability	Challenges the idea of sporadic mtDNA deletions, suggesting nuclear gene defects as a cause
K. Li et al. [31]	m.15897G > A in tRNAThr	Impaired glucose tolerance, average diabetes onset at 57 years	Decreased tRNAThr levels, altered protein synthesis, reduced ATP production	Offers insights into maternally inherited diabetes pathophysiology and potential treatments
Maraş Genç et al. [92]	m.8296A > G	Maternally inherited diabetes mellitus and deafness; cognitive impairment, leukodystrophy, gastrointestinal dysmotility	Pathogenic effect on nuclear-encoded mitochondrial proteins involved in ATP synthesis	Emphasizes the complex relationship between mitochondrial genetics and metabolic disorders
Yang et al. [93]	tRNATrp A5514G, tRNASer (AGY) C12237T	Maternally inherited diabetes and deafness in Chinese family	Potentially pathogenic variants affecting mitochondrial function	Suggests screening for mt-tRNA variants for molecular diagnosis and prevention of mitochondrial diabetes
Urban et al. [94]	m.9143 T > C	Insulin-dependent diabetes, recurrent lactic acidosis, ketoacidosis, immunodeficiency	High heteroplasmy in muscle tissue, variable tissue segregation patterns	Highlights the need to consider mitochondrial disorders in differential diagnosis of diabetes with systemic symptoms
Kyriakidou et al. [95]	m.3243A > G	MIDD, Diabetes and hearing loss in family members	Impaired mitochondrial protein synthesis and OXPHOS	Emphasizes the importance of genetic testing and physical examination in atypical diabetes presentations

**Table 1:** Role mtDNA genetic variations in diabetes mellitus.

Reference	Role of Mitochondrion	Description	Impact on Diabetes Mellitus
Cojocaru et al. [96]	Energy Metabolism	Mitochondria generate ATP through oxidative phosphorylation.	mtDNA variants disrupt ATP production, leading to reduced energy availability and impaired cellular functions, contributing to insulin resistance.
Bhatti et al. [97]	Oxidative Stress Response	Mitochondria manage reactive oxygen species (ROS) production and detoxification.	Increased ROS due to mitochondrial dysfunction causes oxidative damage to cells, promoting insulin resistance and beta-cell dysfunction.
Diane et al. [98]	Insulin Secretion	Mitochondria in pancreatic beta-cells are crucial for insulin secretion.	Genetic varoiation, such as m.3243A > G impair insulin secretion by affecting mitochondrial ATP production and calcium signaling in beta-cells.
D. Li et al. [99]	Mitochondrial DNA (mtDNA) Maintenance	Mitochondria have their own DNA, which is susceptible to variants.	Variants in mtDNA (e.g., m.3243A > G, ND4, tRNA Ala) are linked to mitochondrial diseases, including MIDD and increased susceptibility to type 2 diabetes.
R.-L. Li et al. [70]	Apoptosis Regulation	Mitochondria control programmed cell death.	Mitochondrial dysfunction can lead to inappropriate apoptosis, contributing to the loss of insulin-producing beta-cells.
Zong et al. [100]	Mitochondrial Biogenesis	The process of creating new mitochondria.	Impaired biogenesis due to mtDNA variations reduces the number and functionality of mitochondria, exacerbating metabolic defects.
Fauconnier et al. [101]	Calcium Homeostasis	Mitochondria regulate intracellular calcium levels.	Disrupted calcium handling affects insulin secretion and muscle function including cardiac function, contributing to glucose intolerance and diabetes.
Handy and Holloway [102]	Lipid Metabolism	Mitochondria are involved in fatty acid oxidation.	Impaired fatty acid oxidation due to mitochondrial dysfunction leads to lipid accumulation and insulin resistance.
Takeda et al. [103]	Thermogenesis	Mitochondria generate heat in brown adipose tissue.	Dysfunctional mitochondria reduce thermogenic capacity, leading to decreased energy expenditure and obesity, a risk factor for type 2 diabetes.
Galizzi and Di Carlo [104]	Signal Transduction	Mitochondria participate in cellular signaling pathways.	Altered mitochondrial signaling can disrupt glucose metabolism and insulin signaling pathways, promoting diabetes development.

#### 3.1.1 Regulation of mitochondrial DNA dysfunction in diabetes

Schwartz et al.'s study on *C. elegans* reveals that primordial germ cells (PGCs) regulate by through unique regulation in mtDNA quantity and quality through two mechanisms: cannibalism of PGC lobes combined with general autophagy to reduce total mtDNA quantity, and PINK-1-mediated reduction of mutant mtDNA heteroplasmy to enhance mtDNA quality. This dual regulation ensures the optimal founding population of mitochondria before the germ line expands and differentiates. The reduction in mtDNA quantity resets the mtDNA copy number to around 200, which is actively maintained in germline stem cells (GSCs) as they replicate. PINK-1 plays a role in reducing the fraction of mutant mtDNAs in PGCs, potentially eliminating them from the germ line permanently [105]. Studies by Sarah Weksler-Zangen et al., showed that diabetes mellitus is linked to mitochondrial dysfunction, especially in inherited mitochondrial diseases. The severity of mitochondrial-related diabetes varies depending on the specific variant and the level of affected mitochondria copies. Screening family members for diabetes is crucial, as it can present with distinct features and complications. Managing diabetes in patients with mitochondrial disorders is challenging, as metformin, the first-line treatment for T2DM, is not recommended due to the risk of lactic acidosis. Instead, SGLT-2 inhibitors and mitochondrial GLP-1-related substances are preferred. Evidence implicated that mitochondrial dysfunction as a primary defect in T2DM in humans is limited. Further, recent studies in the Cohen diabetic sensitive (CDs) rat have highlighted the role of the mitochondrial respiratory-chain enzyme cytochrome *c* oxidase (COX) in regulating GSIS. This suggests that islet-COX deficiency is the primary defect causing diabetes. Further research into COX and other mitochondrial factors in diabetes may lead to novel approaches for diagnosing and treating diabetes in patients with mitochondrial diseases and mitochondrial dysfunction [106].

### 3.2 Therapeutic insights

Sodium-glucose cotransporter 2 (SGLT2) inhibitors are widely used for managing diabetes due to their cardiovascular and renal benefits. However, emerging evidence suggests that these agents may exacerbate muscle atrophy and weight loss, particularly in patients prone to sarcopenia [107,108]. SGLT2 inhibitors may increase glucagon levels and promote lipolysis, potentially contributing to muscle wasting by reducing insulin-mediated anabolic effects in skeletal muscle [109,110].

Additionally, mitochondrial dysfunction in muscle cells, exacerbated by diabetes, may compound these effects. Reduced ATP production and increased oxidative stress impair muscle regeneration and repair mechanisms [111]. Incorporating strategies to monitor and mitigate muscle health, such as resistance training and nutritional interventions, alongside SGLT2 inhibitor therapy, could improve patient outcomes. Together, the impacts of mitochondrial dysfunction in diabetes, ranging from retinopathy and macrovascular complications to treatment-related challenges, are crucial for advancing patient care. By exploring the interconnected pathways of mitochondrial health, oxidative stress, and metabolic regulation would provide a comprehensive structure for developing targeted therapeutic strategies and improving clinical outcomes in diabetes management.

### 3.3 Expression studies by mtDNA variants associated with diabetes

Expression studies of mitochondrial DNA (mtDNA) variants associated with diabetes have provided valuable insights into their impact on cellular function. Following are the studies underscore the importance of understanding the expression patterns of mtDNA variants in diabetes and related complications. Studies by Inshah Din et al., investigated the association of a *UCP2* gene polymorphism (−866 G/A) and its expression with diabetic predisposition in the North Indian population. UCP2, a mitochondrial uncoupling protein, is involved in regulating metabolic pathways and has been associated with BMI and hyperinsulinism. The study found that the −866 G/A variant allele of the *UCP2* gene polymorphism (−866 G/A) is significantly associated with T2DM, with the AA genotype showing a 3.45-fold increased risk of diabetes. The *UCP2* gene expression was 4.2-fold lower in diabetic patients compared to controls, indicating a role for UCP2 in T2DM pathogenesis. Older age and higher BMI were associated with further decreases in UCP2 expression and increased risk of T2DM. The study suggests that *UCP2* gene expression and the −866 G/A SNP may play significant roles in T2DM development and progression [112]. Medini et al.'s study on mitochondrial gene expression in pancreatic alpha and beta cells from humans and mice found two distinct populations of human beta cells. These populations showed differences in mitochondrial RNA variantal repertoire and selective signatures, suggesting the presence of two previously unrecognized beta cell populations. In contrast, human alpha cells did not show consistent divergence in mitochondrial gene expression. The findings suggest that pancreatic beta cells exhibit heterogeneity in mitochondrial gene expression, which could impact their metabolic and regulatory functions. Further research is needed to explore these findings for disease conditions like type 2 diabetes and to determine if similar mitochondrial heterogeneity exists in other cell types and tissues [113]. Rong et al. developed a rapid TaqMan-MGB quantitative real-time PCR method for detecting and quantifying the mt.3243 A > G variant in mitochondrial DNA, a factor in diseases like MDD and mitochondrial encephalomyopathy. The method showed high sensitivity and reliable quantification, correlating well with other detection methods. Urinary sediments, leukocytes, or hair follicles could be ideal templates for detecting and quantifying the variant. This method offers a rapid, accurate, and cost-effective approach for detecting mitochondrial variants [114]. Kazuo Tomita et al. developed a sensitive method using double TaqMan probes and qPCR to detect variant frequencies in mitochondrial DNA. The method was designed to detect the A3243G variant associated with MELAS syndrome. The method accurately calculated variant frequencies using FAM and VIC probes. The angle-degree method improved the accuracy of variant frequency determination compared to existing methods. This method could also be applied to other diseases, such as diabetes, indicating its potential for studying mitochondrial variants associated with diabetes [115].

### 3.4 Studies on mtDNA variants with cell culture models

Understanding the cellular and molecular alterations resulting from mtDNA variants is essential for developing effective treatments. Studies have investigated the m.3243 A > G variant, commonly associated with MIDD and mitochondrial encephalopathy, lactic acidosis, and stroke-like episodes (MELAS). These studies used different approaches, such as examining urine-derived stem cells (USCs) and skin-derived fibroblasts, to elucidate the variant's effects and potential therapeutic targets. The findings provide valuable insights into the pathology of mitochondrial diseases and highlight the importance of mitochondrial function in diabetes and related disorders. Few studies were listed to showcase the impact of mtDNA variant in different cell culture model and shown in Table 3.

### 3.5 Next-generation sequencing studies on mtDNA linking diabetes

Next-generation sequencing (NGS) has revolutionised the study of mitochondrial DNA (mtDNA) variants in diabetes and its complications. The following studies demonstrate the importance of NGS in understanding the role of mtDNA variants in diabetes and related disorders. Studies by Materiah Salem Alwehaidah et al. has identified novel non-synonymous and synonymous variants in mitochondrial DNA (mtDNA) variants in Kuwaiti subjects with Psoriasis (Ps), Type 2 Diabetes, and Ps-T2D. The variants were found primarily in genes encoding complex I, III, and V subunits. The patient group causing changes in essential mitochondrial enzyme complexes and RNA components. Most variants were homoplasmic, with some synonymous variants also identified. The study suggests that mtDNA variants may contribute to the pathogenesis of Ps, T2D, and Ps-T2D, highlighting the need for further functional analysis to understand their role in these diseases. ^51^

Studies by Hyun-Wook Chae et al., investigated the link between mitochondrial myopathy, encephalopathy, lactic acidosis, and stroke-like episodes (MELAS) syndrome and diabetes in children and young adolescents. Researchers used next-generation sequencing to analyze mitochondrial DNA (mtDNA) of 32 individuals diagnosed with MELAS syndrome and the mtDNA A-to-G transition at nucleotide 3243. The study found an inverse correlation between variantal load and the onset of MELAS syndrome symptoms, age at diagnosis, and the presence of diabetes. Furthermore, Insulin resistance or sensitivity indices did not significantly differ between low and high variant load groups. Over a 3.7-year follow-up period, insulin resistance indices remained stable, with no significant difference between baseline and follow-up. In conclusion, the study suggests that variantal load in MELAS syndrome is associated with the onset of symptoms and related diseases, such as mitochondrial diabetes, but it does not appear to influence the disease progression. The findings suggest that variantal load in MELAS syndrome may play a role in the development of these conditions in children and young adolescents. [81] Studies by Kevin Colclough et al. evaluated routine testing of syndromic diabetes genes in patients with suspected maturity-onset diabetes of the young (MODY) by Next Generation Sequencing (NGS). The study found that one in five patients with suspected MODY had a variant in a syndromic diabetes gene, despite lacking typical features. The study emphasized the importance of expanding testing to include syndromic genes, as overlapping diabetes features with MODY often lead to referrals for genetic testing. Variants in syndromic diabetes genes accounted for 19 % of all monogenic diabetes cases, with the mitochondrial m.3243 A > G variant and HNF1B variants being the most common. The study suggests routine testing of syndromic monogenic diabetes genes, particularly m.3243 A > G and HNF1B, in patients with suspected MODY who do not exhibit typical genetic syndrome features. [82].

The study by Jiang et al. investigated the inheritance patterns of the m.3243 A > G variant in pedigrees with MIDD syndrome. They found that the variant occurred de novo in one family, indicating it can happen without maternal inheritance. The proband and her son both had diabetes, mild bilateral hearing loss, abnormal brain MRI, and impaired mitochondrial function. Whole mitochondrial DNA sequencing revealed an additional heteroplasmic substitution at m.16093 T > C, which has not been previously reported in MIDD or related syndromes [83].

A study by Shand et al. examined the mtDNA variant mt .3243 A > G, linked to maternally inherited diabetes and deafness. The researchers found that monozygotic twins carrying the variant developed end-stage renal disease (ESRD). The twins had a higher heteroplasmy load, developing ESRD 15 years earlier than her sister. This suggests a correlation between heteroplasmy level and the age at which ESRD develops in patients with mt.3243 A > G-related kidney disease. The study also observed the progression of mt.3243 A > G variant-related renal disease in the twins, suggesting that the levels of peripheral blood mt.3243 A > G heteroplasmy reflected in renal tissue heteroplasmy may explain the difference in disease progression. The findings could have implications for understanding the pathogenesis of these conditions and developing targeted therapies [84].

The study by Yagi et al. examined the link between the mitochondrial DNA m.3243 A > G variant, which causes mitochondrial encephalopathy, lactic acidosis, and stroke-like episodes (MELAS), and its associated multi-organ disorders, including diabetes. The researchers used genetic and pathological examinations from autopsied subjects to understand the relationship between the variant's organ heteroplasmy and clinical phenotypes, specifically looking at the age at death. They found that high heteroplasmy levels of the m.3243 A > G variant were found in non-regenerative organs like the brain, myocardium, and endocrine glands, while low levels were found in regenerative organs like the bone marrow, spleen, and peripheral leukocytes. Liver heteroplasmy was found to be high despite being a regenerative organ. The study also found that the age at onset of MELAS-associated symptoms significantly contributed to an early age at death, and higher organ heteroplasmy levels correlated with a lower age at death. This suggests that the degree of heteroplasmy, particularly in the liver, is a key factor in determining the age at death in individuals with the m.3243 A > G variant [116]. The study by Eirini Kaisari et al. investigated the link between the mtDNA A3243G point variant and diabetes-related complications, specifically retinopathy in female patients. The A3243G variant is associated with various disorders, including MELAS, MID, and CPEO. The study involved six female patients aged 37 to 70 years with the variant, who exhibited maculopathy. The maculopathy was not related to age and progressed slowly. The study concluded that the A3243G variant can lead to various clinical presentations, including maculopathy, which can impact central visual function from asymptomatic to legal blindness. The findings should prompt ophthalmologists to complete their personal and family history, including diabetes mellitus and deafness [117].

Marco-Campmany et al.'s showed a case of MIDD in a 49-year-old man with a mitochondrial variant. The patient developed macular cysts 20 years after the initial presentation of his macular dystrophy. Intravitreal bevacizumab treatment resolved the cystic changes and restored visual acuity. The study emphasizes the importance of diagnosing MIDD to prevent complications like macular dystrophy and cysts. Further research is needed to understand the pathogenesis and treatment of this complication [118]. A study in northeast India examined the role of mtDNA variants in the development of type 2 diabetes in a specific ethnic tribe. Sequencing of the mitochondrial genomes of diabetic patients identified mtDNA variants and their association with familial T2D. They found that certain mtDNA variants, such as 8584 G > A and 10398 A > G in the *ND3* gene, were significantly associated with T2D risk. A novel frame-shift substitution, ND5: 81_81ins A at position 12,417 was observed and found to be possible target for diabetes and provides valuable insights into the role of mitochondrial variants in T2D susceptibility in the Mizo population [119]. Barbara Lombardo et al.'s study on two brothers with a multisystemic disorder, Alstrom syndrome including diabetes, found a definitive diagnosis through mitochondrial DNA sequencing via whole exome sequencing. The variant in the *ALMS1* gene, which affects intracellular trafficking, ciliary function, and insulin receptor trafficking, caused a frameshift at the protein level and a premature stop codon. This highlights the importance of genomic analysis in diagnosing rare complex diseases and the role of WES in providing definitive diagnoses and guiding personalized treatments. [120] A study by Ohwada et al. found that a patient with mitochondrial disease and diabetes mellitus experienced a transient increase in blood lactate levels during insulin infusion therapy. This increase was not due to worsening heart failure or kidney function, but rather due to enhanced glycolysis in insulin-sensitive tissues with mitochondrial dysfunction and decreased lactate consumption in skeletal muscle and the failing heart. The study suggests that factors like tissue hypoxia or uncoupling between glycolysis and mitochondrial oxidation can lead to increased lactate production even under aerobic conditions [121].

### 3.6 Case/familial studies on mtDNA variants linking diabetes

Case and familial studies provide valuable insights into the genetic basis and clinical manifestations of these disorders. By examining individual cases and families with mitochondrial disorders, researchers can identify specific mtDNA variants associated with disease and gain a better understanding of their impact on mitochondrial function and cellular metabolism. Additionally, these studies help elucidate the complex relationship between mitochondrial dysfunction and diabetes, providing important implications for diagnosis, management, and potential therapeutic interventions. A recent rare case report describes a neonatal mitochondrial disease presentation in an infant born to a mother with diabetes. The infant had hypertrophic cardiomyopathy, a rare condition in neonatal mitochondrial disease and typically with poor prognosis. The mother had maternal diabetes, which can cause hypertrophic cardiomyopathy in infants. The infant's persistent ventricular hypertrophy was diagnosed as mitochondrial disease associated with the m.3243 A > G variant in a mitochondrial tRNA leucine 1 gene. This case highlights the need for clinicians to consider mitochondrial disease in cases of unexplained HCM, especially in infants born to mothers with diabetes. Early recognition and diagnosis of mitochondrial disease can significantly impact management and prognosis [122]. A 64-year-old woman with mitochondrial cardiomyopathy and maternally inherited diabetes and deafness underwent transcatheter edge-to-edge repair (TEER) with the Mitra Clip system, reducing her functional mitral regurgitation. Despite medical therapy and taurine supplementation, she had recurrent heart failure and was high risk for surgery. The study highlights the use of TEER as a less invasive and effective treatment for severe MR in patients with mitochondrial disease, suggesting it may be a feasible option for these patients [123]. Studies showed that the synthesis and characterization of the anticodon stem-loop of human mitochondrial tRNA for methionine (hmt-tRNAMet), focusing on the effects of disease-related variants at position 37 (A4435G), which are linked to severe mitochondrial disorders like hypertension, type 2 diabetes, and LHON [124].

Mitochondrial DNA m.3243 A > G variant in a Taiwanese cohort of cerebellar ataxia patients with unknown genetic diagnosis, focusing on its association with diabetes mellitus. Two patients with the variant showed slowly progressive ataxia, diabetes mellitus, and hearing impairment. Neuroimaging studies revealed cerebellar involvement in both patients, suggesting the variant may contribute to cerebellar ataxia in diabetes patients, emphasizing the importance of mitochondrial dysfunction in neurodegenerative disorders [125].

The studies showed that genetic characteristics of Han Chinese families with the m.3243 A > G variant in the mitochondrial *tRNALeu* gene, focusing on its association with diabetes mellitus. Identified seven families with varying phenotypes, including mitochondrial encephalomyopathy, lactic acidosis, stroke-like episodes, life-threatening mitochondrial myopathy, and neuropathy, ataxia, and retinitis pigmentosa. The study also explored the variants and its association with different haplogroups focusing on nuclear factors associated with the disease [126]. The pathogenic role of the tRNAGly T10003C variant in diabetes mellitus (DM) was evaluated by conducting conservation assessments across species and used bioinformatics analysis to predict the secondary structure of mt-tRNAGly in both wild-type and mutant versions. They screened 500 unrelated DM patients and 300 healthy controls for the presence of the T10003C variant. The findings showed that the T10003C variant was not highly conserved and did not cause a change in the secondary structure of mt-tRNAGly. The study emphasizes the importance of careful assessment of mtDNA variants in understanding their role in the disease pathogenesis [127]. Recent case or familial studies linking mtDNA variant with diabetes are tabulated in Table 4.

### 3.7 mtDNA variants linking other diseases

Mitochondrial DNA variants are known to be associated with a wide range of diseases beyond diabetes. These variants can affect various organs and systems in the body, leading to diverse clinical manifestations. Diseases linked to mtDNA variants include neurodegenerative disorders, such as Alzheimer's disease and Parkinson's disease, as well as neuromuscular disorders like mitochondrial myopathy. Additionally, mtDNA variants have been implicated in cardiovascular diseases, certain types of cancer, and even aging-related processes. Understanding the role of mtDNA variants in these diseases is crucial for developing effective diagnostic and therapeutic strategies.

Max Borsche et al.'s study on Parkinson's disease (PD) found a higher ratio of gluconeogenesis / glycogenolysis (GNG/GP) in idiopathic PD patients compared to healthy controls, possibly due to increased glycolysis in their blood cells. The study also discussed the potential role of mitochondrial dysfunction in PD, suggesting that increased glycolysis might be the body's response to mitigate insufficient ATP production resulting from mitochondrial dysfunction. Although epidemiologic studies on the association between diabetes and *PRKN* gene alterations are limited, the study noted an increased frequency of diabetes in heteroplasmy PRKN variant carriers. However, there was no significant difference in GNG in GP for biallelic PRKN variant carriers compared to healthy controls. The findings support different pathogenic mechanisms between IPD and PRKN-PD, suggesting different biochemical principles of the link between mitochondrial dysfunction and alterations in glucose metabolism in PD [128].

The studies evaluated the mitochondrial DNA variant m.3243 A > G on the inner ear in individuals with MIDD and MELAS syndromes. Eight subjects with the variant had sensorineural hearing loss (SNHL), with varying severity. Speech discrimination scores indicated different types of SNHL. Video head impulse tests detected pathology in semicircular canals, and vestibular-evoked myogenic potentials were absent in most subjects. The study concluded that individuals with the m.3243 A > G variant experience dysfunction in both auditory and vestibular systems [129]. Tran et al. highlights the link between mitochondrial diseases and diabetes, especially in the context of MELAS syndrome. Diabetes is a common endocrine manifestation of mitochondrial diseases, presenting with varying phenotypes resembling type 1 or type 2 diabetes. The onset of mitochondrial diabetes can be latent or acute, leading to rapid cognitive decline in patients with MELAS syndrome. The article emphasizes the importance of recognizing and monitoring diabetes in patients with mitochondrial diseases, as it can significantly impact their clinical course and quality of life. Clinicians should be aware of the potential for acute hyperglycemic crises in these patients [130]. A study by Saeed et al. found that diabetic women with Polycystic Ovary Syndrome (PCOS) and diabetes have higher levels of certain hormones and insulin resistance markers. The study identified ten variants in mt-tRNA genes, mainly in conserved regions. The lower mtDNA copy numbers in the PCOS group suggest mt-tRNA variants may contribute to PCOS through mitochondrial dysfunction and insulin resistance, underscoring the importance of mitochondrial genetics in the disease pathogenesis [131]. Mitochondrial dysfunction and its potential role in gestational diabetes mellitus were evaluated and showed reduced placental mitochondria may lead to impaired placental development and increased risk of T2DM in offspring. Furthermore, mitochondrial dysfunction affects age-related decline in female fertility, with conditions like polycystic ovarian syndrome (PCOS) and primary ovarian insufficiency affecting oocyte quality and fertility. Therapeutic strategies like coenzyme Q10 and antioxidant cocktails have shown promise in improving mitochondrial function, but challenges remain, particularly regarding the transfer of functional mitochondria or cytoplasm, requiring further study for clinical application [132].

Several mtDNA variants mentioned in the MITOMAP database and numerous literature reports have high population frequencies. However, variants are not pathogenic by traditional standards and are still relevant to diabetes by other complex mechanisms. These mtDNA variants in various populations prompt the authors to hypothesize that beyond primary pathogenicity, factors such as context-dependent effects, modifier roles, and population-specific factors contribute to their contribution to diabetes pathogenesis. For instance, the mutations in ND4 or tRNA genes were found to have differential effects on insulin release and resistance in different populations, indicating that the functional impact of these variations might be modified by genetic background and environmental exposures and increase susceptibility to diabetes under certain conditions (M. [32,133]).

In addition, we require more directed functional and epidemiological research to clarify these receptors' roles. Some studies have already suggested that patients with the same variant show different clinical presentations, which could be due to epigenetic mechanisms, interaction with nuclear DNA, and even metabolic stressors such as a high-fat diet. For example, heteroplasmic mutations of the ND4 and tRNA genes were associated with high levels of ROS that have caused oxidative damage and diminished insulin signaling only in a subset of populations where such mutations reach higher frequencies [6,134] It was suggested that the functional and epidemiological investigations. Studies should be conducted in deep detail to identify precisely under what conditions these variants become pathogenic, thus again expanding knowledge of mitochondrial-related variants in disease and enabling precision in medicine for mitochondrial diseases.

## 4 Conclusion

Mitochondrial DNA variants in diabetes are critical for developing tailored therapeutics that address mitochondrial dysfunction and improve patient outcomes. This thorough study aims to improve our understanding of mitochondrial biology in diabetes, paving the path for novel therapeutic approaches. Understanding the role of mitochondrial dysfunction in diabetes is critical for designing targeted medicines that increase mitochondrial activity and may prevent or treat diabetes. More research is needed to determine the precise pathways driving mitochondrial dysfunction in insulin resistance and diabetes. These findings shed light on the complex link between mitochondrial malfunction and diabetes, emphasizing the importance of mtDNA variants in metabolic regulation and disease progression. These findings highlight the possibility of targeted mitochondrial therapy to enhance diabetic outcomes. The influence of mitochondrial DNA (mtDNA) variants on cellular function and disease development in diabetes is highlighted by expression studies. The fact that these variants are associated with a variety of disorders highlights the need for additional study to comprehend their functions and create efficient diagnostic and treatment plans. Important research highlights the broad clinical implications of mtDNA variants in metabolic and neurological illnesses. The pathophysiology of diabetes and mitochondrial function are significantly impacted by mtDNA variants, as evidenced by animal studies, which also point to possible treatment targets. These models improve our knowledge of diabetes and its complications by offering insightful information about molecular pathways such as ALDH2*2 variants, Cdk5 expression, and CRIF1 lack. Diabetes pathophysiology is greatly impacted by mtDNA abnormalities, which also affect the severity of the disease and mitochondrial function. These variants and their part in the disease are explained using sophisticated tools such as NGS and cell models, which provide prospective treatment targets and better approaches to managing the condition. Additional case and family studies demonstrate the complex connection between diabetes and mitochondrial dysfunction, emphasizing genetic analysis and its role in diagnosis and treatment. Certain mtDNA variants, like m.3243 A > G, are important in mitochondrial illnesses and frequently manifest as various clinical symptoms. Providing customized therapeutic measures, early detection, and thorough genetic testing can greatly enhance patient outcomes.

## CRediT authorship contribution statement

**Praveen Kumar K.S.:** Writing – review & editing, Writing – original draft, Validation, Supervision, Resources, Methodology, Funding acquisition, Formal analysis, Data curation, Conceptualization. **M.N. Jyothi:** Writing – review & editing, Writing – original draft, Validation, Methodology, Formal analysis, Data curation, Conceptualization. **Akila Prashant:** Writing – original draft, Visualization, Validation, Supervision, Methodology, Formal analysis, Conceptualization.

## Declaration of competing interest

Authors declare that they do not have any conflict of interest.

## Data Availability

Data will be made available on request.
